# Algorithm Aversion Towards AI-Generated Multiple-Choice Questions

**DOI:** 10.1007/s40670-026-02866-6

**Published:** 2026-08-28

**Authors:** Matthias Carl Laupichler, Susanne Eigster, Seifollah Ahmadi, Ilona Grunwald Kadow, Tobias Raupach, Johanna Flora Rother

**Affiliations:** 1 Institute of Medical Education, University Hospital Bonn, Venusberg-Campus 1, 53127 Bonn, Germany; 2 Institute of Physiology II, Department of Medicine, University of Bonn, Bonn, Germany

**Keywords:** Artificial intelligence, Algorithm aversion, Anti-AI bias, Multiple-choice question, AI literacy, Large language model

## Abstract

Algorithm aversion and anti-AI bias describe the human tendency to evaluate decisions made by human agents more favorably than those made by artificial intelligence (AI) algorithms. This study aimed to examine whether algorithm aversion influences students’ evaluations of multiple-choice questions (MCQs) as well as the psychometric quality of items generated by a large language model (LLM). Additionally, the authors explored the possible influence of AI literacy and attitudes toward AI. The research team administered a formative examination consisting of two blocks. Block 1 was labeled “authored by a medical educator,” although 50% of the 20 items were in fact generated by an LLM. Block 2 was labeled “generated by an LLM,” but likewise included 50% human-authored items. The human-authored and AI-generated questions were based on the same 20 learning objectives. Following each item, medical students rated its perceived relevance for exam preparation and its quality of wording. AI literacy and attitudes toward AI were assessed using validated instruments. Participants solved significantly more LLM-generated items (*M* = 77%) correctly than human-authored items (*M* = 48%), *U* = 34, *p* < .001, *r *= 0.71. No significant differences were observed in discriminatory power. Cumulative link mixed models revealed that students rated the relevance of actually AI-generated items more positively. In addition, they exhibited an anti-AI bias in their ratings of item wording quality, as items labeled as LLM-generated were rated significantly worse, irrespective of their actual origin. Although the overall findings statistically suggest an anti-AI bias, their practical implications remain unclear, as relevance and wording quality were rated very positively overall.

**Supplementary Information:**

The online version contains supplementary material available at https://doi.org/10.1007/s40670-026-02866-6.

## Introduction

Artificial intelligence (AI) and in particular, Large Language Models (LLMs) such as OpenAIs ChatGPT, have become one of the most widely discussed areas of research in recent years [1, 2].

Several international studies in the last few years have investigated the abilities and limitations of ChatGPT in the context of education in general [3, 4] and medical education in particular, for example demonstrating that ChatGPT was able to pass important medical examinations from several different countries, such as the United States Medical Licensing Exam (USMLE) [5].

Another way to employ LLMs is by using them to prepare exam questions, which could save valuable time, financial and personnel resources [6]. A 2023 study compares the item parameters of human-made multiple-choice-questions (MCQs) with those generated by ChatGPT 3.5 [7]. The questions were presented without a label and the participants were asked to guess which questions were created by ChatGPT and which questions were human-made. The results demonstrated no significant difference in item difficulty, but the human-made MCQs showed a higher discriminatory power (i.e., a question’s ability to tell the difference between high- and low-performing test-takers), compared to the questions made by ChatGPT. Interestingly, the results also showed that only 57% of the questions were identified correctly as either human- or ChatGPT-made. Following this pilot study, other research groups have conducted comparable investigations [8–10].

Creating high quality questions with ChatGPT does not only benefit educators when preparing materials and exams [11], but it can also benefit students through the so called testing effect [7], which implies that taking a test about learning material and therefore testing one’s own memory improves the long-term retention of the materials [12]. This concept has been successfully employed to improve students’ learning outcomes [13, 14], suggesting that the students might also benefit from quick and easy tools to create questions, which they can use to test their own knowledge. In our study, multiple-choice questions are accordingly conceptualized as learning tools rather than mere assessment instruments.

Following the continuous release of increasingly advanced and capable LLM versions, recent studies have adapted the approach to compare AI-generated MCQs based on newer LLM models with human-made MCQs and their results suggest that since our previous study [7], the LLM has improved its ability to generate high-quality questions [15, 16].

Previous research has introduced the term *algorithm aversion*, which describes the concept of humans preferring human-made judgments and decisions to those made by a mathematical algorithm [17]. Recently, a German study investigated this concept in the context of medical information and found that participants rated medical advice as significantly less reliable and less empathic when the content was labeled as “AI”- or “AI & human”-generated. The participants also indicated that they were less willing to follow the advice when AI was seemingly involved in the process [18]. Interestingly, the participants all received the same advice with the only difference being the label, suggesting that humans in some cases do not trust AI-generated content. Therefore, even though the opposite term *algorithm appreciation* has also been discussed in previous research [19], the general literature seems to lean towards an anti-AI bias in the general population [20].

A possible categorical negative bias toward AI-generated MCQs could substantially limit students’ acceptance of such questions, which in turn might adversely affect the use of novel, AI-supported learning resources [21]. Accordingly, the primary aim of this study was to examine whether medical students evaluate MCQs purportedly generated by ChatGPT more negatively in terms of their perceived relevance to individual learning outcomes and the quality of their wording compared to questions authored by medical educators.

Beyond a general, intrinsic tendency toward algorithm aversion, interindividual differences may also play a role in the evaluation of AI-generated educational materials. In this context, particular attention has been given to students’ general competencies in dealing with AI [22], commonly referred to as AI literacy [23], as well as their attitudes toward AI [24, 25]. Therefore, a secondary aim was to explore the extent to which students’ AI literacy and attitudes towards AI influence their evaluations of the MCQs.

Regarding the item-parameters, we expected the label to have no influence on the item difficulty and discriminatory power, since these are not measured through self-report. Nonetheless, based on our previous study [7], we expected questions that were in fact generated by an AI-system to exhibit significantly lower discriminatory power.

### H1

AI-generated MCQs have significantly lower discriminatory power than MCQs developed by a human medical educator.

Although findings from studies in other domains might suggest the presence of an anti-AI bias toward AI-generated learning materials such as MCQs, we limited ourselves to formulating non-directional, exploratory research questions because robust evidence in this specific context is still lacking:

### RQ1

Does the label, the actual origin of the question, or their interaction have a statistically significant effect on the perceived relevance of the question or the perceived quality of its wording?

In addition, we wanted to examine which possible variables could influence these relationships and therefore investigated the following research question:

### RQ2

Does a person’s self-reported AI literacy or attitudes towards AI have a statistically significant moderating effect on the influence of label on the perceived relevance or perceived quality of wording of an MCQ?

## Materials and Methods

### Study Design & Instruments

We employed a within-subjects field experimental design. For this purpose, we developed a formative examination in the medical subject of neurophysiology, comprising 40 single-best-answer MCQs, each with five answer options. Of these, 20 items were authored by a faculty member specialized in neurophysiology at Bonn Medical School, while the remaining 20 were generated by a large language model (OpenAI’s ChatGPT, version 4o). Both sets of questions were based on the same 20 core neurophysiology topics (Supplementary Material S1) to ensure content comparability. The LLM-generated items were reviewed by a neurophysiologist experienced in developing MCQs to verify their content accuracy. The human medical educator’s review was limited to verifying the accuracy of the questions and answer options; no analysis of the anticipated difficulty or evaluation of the relevance of the questions was conducted. Since all LLM-generated MCQs were deemed correct, no subsequent revisions or adjustments were required. The prompt used for AI-based question generation is provided in Supplementary Material S2. All questions were composed in German.

**Fig. 1:**
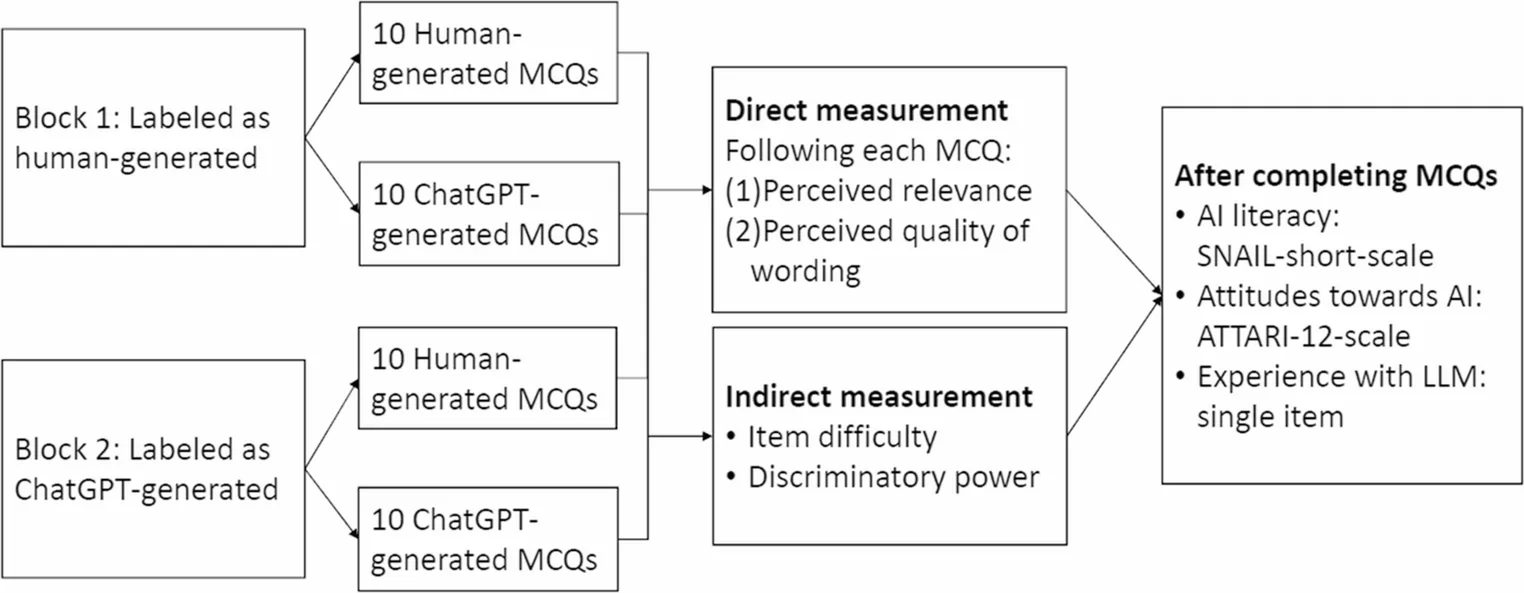
Study design

Subsequently, we assigned 10 questions from each set randomly to one of two blocks. The first block was labeled as containing questions written by a medical educator from Bonn Medical School. In reality, only half of these items were human-generated; the other half were created by ChatGPT. Conversely, the second block was labeled as consisting of questions generated by ChatGPT, although a human expert had developed half of the items in this block. In addition, we randomized the order of questions within each block (see Fig. 1).

Following each MCQ, we asked participants to respond to two evaluation items: [1] how relevant they perceived the question to be for preparing for their upcoming mandatory summative examination (“This question appears relevant for examination preparation.”), and [2] how well-formulated they considered the question to be (“This question is precisely and appropriately formulated.”. Both items were rated on a 7-point Likert scale (1 = “Strongly disagree” to 7 = “Strongly agree”).

After completing the MCQ items, participants’ AI literacy was assessed using a validated German short form of the 12-item SNAIL instrument [14]. SNAIL comprises three subscales representing core components of AI literacy: *Technical Understanding*, *Critical Appraisal*, and *Practical Application*. Subsequently, participants’ attitudes toward AI were measured using the ATTARI-12 scale [26], which includes 12 items assessing cognitive, affective, and behavioral dimensions of AI-related attitudes (see Fig. 1 for a visual representation of the study design). Finally, we measured participants’ prior experience with LLMs with a single, self-developed, six option ordinal-scaled item (see Supplementary Material S3 for the questionnaire as well as Supplementary Material S4 for an English version of a selection of sample MCQs).

### Procedure & Participants

We invited all students enrolled in the neurophysiology course during the summer term of 2025 (*n* = 380) to participate in a formative exam, which served the purpose of preparing them for the actual exam and providing feedback on their current performance levels. The students were informed by email about the formative examination, which was conducted in place of a regular lecture session without mandatory attendance. The students had already been introduced to the 20 topics during previous teaching sessions; therefore, a basic level of knowledge could be assumed. The incentive for participation was the provision of feedback on their individual test performance. The students had 60 min to answer all questions, and both question sets, along with the accompanying questions (e.g., those assessing AI literacy), were completed during a single session. All data were collected using a single paper-and-pencil format questionnaire, on June 27, 2025.

All participants were second-year medical students at Bonn Medical School. In the interest of data parsimony, we collected no additional sociodemographic variables, as we did not posit any hypotheses related to individual-level characteristics. A total of 145 students participated in the study.

The institutional ethics review board of the University of Bonn approved the study (Approval Number: 2025-71-BO).

### Data Analysis

We used the Evasys software (evasys GmbH, Lüneburg, Germany, 2025, Version 10.0) for questionnaire input and processing. We used R (R Core Team, Vienna, Austria, 2025, Version 4.5.0), RStudio (Posit Software, Boston, USA, 2025, Version 3.6.0), and Microsoft Excel (Microsoft Corporation, Redmond, USA, 2025, Version 2025) for data analysis.

As part of the statistical analysis, we calculated the average item difficulty for each MCQ as the proportion of correct responses relative to the total number of responses for that item. Discriminatory power was assessed using the point-biserial correlation between individual item performance and the part-whole corrected total test score. Because we did not expect item parameters to differ based on the assigned label, we examined only the effect of the actual origin of the two MCQ sets. Mean item difficulty and discriminatory power between AI-generated and human-made items were compared using t-tests. Because, based on the results of the Shapiro–Wilk test, the assumption of normality was violated for discriminatory power, a Mann–Whitney U test was conducted as a more robust alternative for both variables (item difficulty and discriminatory power) to ensure the robustness of the analysis.

We used cumulative link mixed models (CLMMs) with a logit link function to analyze RQ1 and RQ2. Separate models were estimated for perceived item relevance and wording quality, both assessed on seven-point ordinal response scales. CLMMs were selected because they accommodate the ordered categorical nature of the outcomes and account for the non-independence of the repeated item-level ratings provided by each student.

For each outcome in RQ1, the model included actual item origin, presented label, and their interaction as fixed effects. A random intercept for participant ID was included to account for individual differences in overall rating tendency across the 40 items. Human-generated origin and the human-generated label served as reference categories. Thus, the interaction term tested whether associations between the presented label and students’ ratings differed depending on the item’s actual origin. Ratings with missing outcome data were excluded separately for the respective model using complete-case estimation. Model convergence was assessed using the maximum absolute gradient and the condition number of the Hessian matrix.

The analysis of RQ2 largely followed the same procedure as the analysis of RQ1. The main difference was that the model formula additionally included AI literacy and attitudes toward AI as potential explanatory variables. Moreover, the analysis focused exclusively on the main and interaction effects of the label and the respective potential explanatory variable, as including actual item origin led to convergence problems and was not central to the hypothesis. Consequently, a separate cumulative link mixed model (CLMM) was fitted for each explanatory variable to facilitate interpretation. Accordingly, four models were calculated: two outcome variables × two potential explanatory variables. An example model formula is:

$$\begin{array}{c}Perceived\;relevance\;\sim\;label\;\times\;\\z-stan dardized\;AI\;literacy\;score\;+\;\left(1\;\left|\;Subject\;ID\right.\right)\end{array}$$

Initially, we had also intended to examine the ordinal variable “experience with using LLMs” as a potential moderator. However, the analysis revealed convergence issues and poor model fit. We therefore decided to report this variable descriptively only and not include it as a fixed effect in the CLMMs.

## Results

### Descriptive Results

**Fig. 2:**
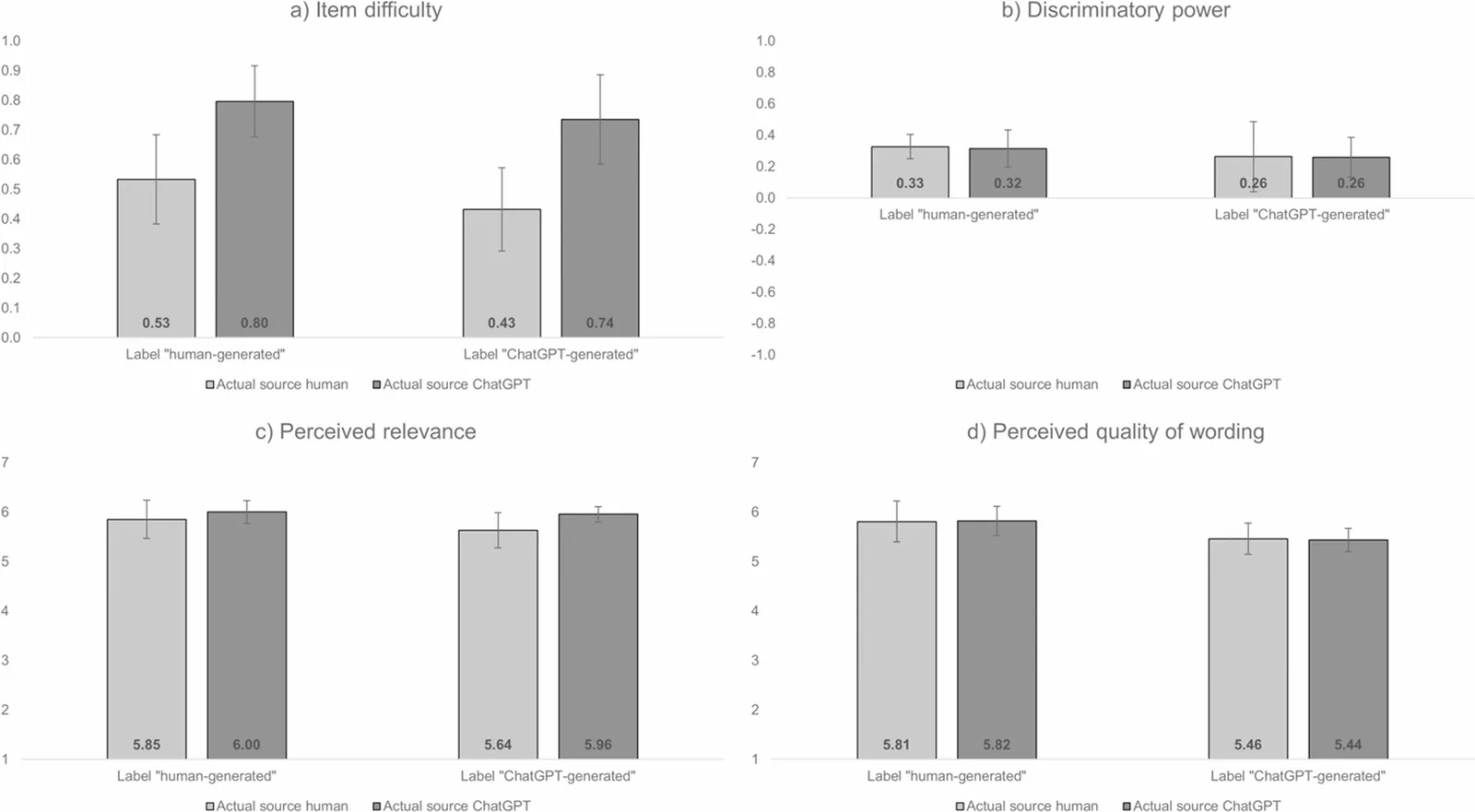
Mean item difficulty, discriminatory power, perceived relevance, and perceived quality of wording, stratified by actual item origin and assigned label. Note The Y-axis limits in the diagrams reflect the full range of theoretically possible values. Item-difficulty values of 0 indicate that all test takers answered the question correctly, meaning that the MCQ was particularly easy, whereas a difficulty value of 1 indicates that no test taker answered the question correctly, meaning that the MCQ was particularly difficult. In general, item-difficulty values are expected to fall approximately between 0.40 and 0.80. A discrimination value of 1 indicates that performance on the individual item is perfectly positively correlated with performance on the remaining test items. A value of 0 indicates no correlation, whereas a value of − 1 indicates a perfectly negative correlation between the item and the remaining test items. Ideally, the discrimination value should be as high as possible, with values above 0.30 often considered acceptable. The scales assessing perceived relevance and perceived quality of wording were operationalized such that higher scores reflected more positive evaluations of the respective constructs

On average, participants correctly answered 9.6 or 48% (standard deviation [SD] = 3.0) of the questions authored by a medical educator, compared to 15.4 or 77% (SD = 2.7) of the questions generated by ChatGPT. The mean discriminatory power of the human-generated items was 0.30 (SD = 0.17), while that of the ChatGPT-generated items was 0.29 (SD = 0.12) (see Fig. 2a and b).

Descriptive statistics for participants’ AI literacy, attitudes toward AI (ATAI), and experience with LLMs are presented in Table 1. The findings indicate that the students rated their AI literacy relatively highly in the domains of Practical Application and Critical Appraisal, with scores approximately 1.2 points above the midpoint of the 7-point Likert scale. In contrast, they rated their Technical Understanding of AI relatively low, with scores on average 1.7 points below the scale midpoint. The students’ attitudes toward AI appear to be differentiated and, on average, were centered around the scale midpoint, although a slight trend toward more positive attitudes was observed. On average, participants reported substantial experience with AI applications, with a median score of 5 on the ordinal 6-point scale.

Descriptive statistics for perceived relevance and quality of wording are presented in Fig. 2c and d.

**Table 1:** Mean, median, and standard deviation for AI literacy, attitudes toward AI, experience with LLMs, and the respective subscales of the assessment instruments

Variable Scores	M	Md	SD
AI literacy (overall score)	4.22	4.33	0.998
Technical understanding (subscale)	2.30	2.00	1.239
Critical appraisal (subscale)	5.20	5.50	1.445
Practical application (subscale)	5.17	5.25	1.185
Attitudes towards AI (overall score)	4.62	4.76	0.958
Cognitive ATAI (subscale)	4.67	4.75	1.054
Affective (subscale)	4.53	4.50	1.159
Behavioral (subscale)	4.66	4.75	1.106
Experience with using LLMs (single item)	4.93	5.00	1.137

*Note*. All values were calculated based on *n* = 140 participants, with the exception of the item assessing experience with LLMs, which was answered by *n* = 134 participants. AI literacy and attitudes towards AI were assessed using a 7-point Likert scale. LLM experience was assessed using an ordinal-scaled multiple-choice question containing six options

### Item Parameters

AI-generated items showed significantly lower difficulty than human-authored items (U = 34, *p* <.001, *r* =.71). There was no significant difference in item discrimination between AI-generated and human-authored items (U = 225, *p* =.51, *r* =.11) (see Fig. 2).

### Rating of MCQs

For perceived relevance, the CLMM revealed a significant interaction between actual question origin and the presented label, *b* = 0.27, *SE* = 0.10, *z* = 2.59, *p* =.010, OR = 1.31. Thus, the association between actual origin and perceived relevance differed depending on whether questions were labelled as human-generated or AI-generated. When questions were labelled as human-generated, items that were actually AI-generated received significantly higher relevance ratings than items that were actually human-generated, *b* = 0.19, *SE* = 0.08, *z* = 2.47, *p* =.014, *OR* = 1.20. The corresponding Holm-adjusted pairwise comparison remained statistically significant, *p* =.041.

The positive interaction indicated that the difference between actually AI-generated and actually human-generated questions was larger when items were labelled as AI-generated. The estimated origin effect in the AI-generated label condition was *b* = 0.46 on the latent log-odds scale, corresponding to an odds ratio of 1.58. Therefore, across both label conditions, actually AI-generated questions tended to receive higher perceived relevance ratings than actually human-generated questions; however, this origin-related difference was more pronounced when questions carried an AI-generated label. The model included a random intercept for students, indicating substantial between-student variability in overall relevance ratings, *σ*^*2*^ = 2.18, *SD* = 1.48. The analysis was based on 5,612 non-missing ratings from 145 students; 188 ratings were excluded because of missing relevance data.

In other words, the perceived relevance of genuinely human-authored MCQs was somewhat lower than that of genuinely ChatGPT-generated MCQs, whereas the “ChatGPT-generated” label led to slightly lower relevance ratings for the MCQs, regardless of whether the items had actually been generated by ChatGPT or authored by a human.

For perceived wording quality, the CLMM showed no significant interaction between actual question origin and presented label, *b* = 0.02, *SE* = 0.10, *z* = 0.18, *p* =.861. Actual question origin was also not significantly associated with perceived wording quality, b = −0.08, SE = 0.07, z = −1.07, *p* =.286, indicating no evidence of a difference in perceived wording quality between questions that were actually AI-generated and those that were actually human-generated.

In contrast, the presented label was significantly associated with wording ratings. Questions labelled as AI-generated received lower perceived wording-quality ratings than questions labelled as human-generated, 𝑏 = −0.55, *SE* = 0.07, *z* = −7.45, *p* <.001, *OR* = 0.57. As the origin-by-label interaction was not significant, this negative association of the AI-generated label with wording ratings was comparable for actually AI-generated and actually human-generated questions.

The model included a random intercept for students, indicating substantial between-student variability in overall relevance ratings, *σ*^*2*^ = 2.34, *SD* = 1.53. The analysis was based on 5,616 non-missing ratings from 145 students; 184 ratings were excluded because of missing relevance data.

### Effect of AI Literacy and Attitudes Towards AI

Self-reported AI literacy was not significantly associated with overall perceived relevance ratings, *b* = 0.17, *SE* = 0.13, *z* = 1.29, *p* =.198. Crucially, AI literacy did not significantly moderate the association between the presented label and perceived relevance, *b* = −0.01, *SE* = 0.05, *z* = −0.26, *p* =.796. Accordingly, there was no evidence that students with higher self-reported AI literacy responded differently to the labels “AI-generated” versus “human-generated” when evaluating question relevance. The model included 5,463 non-missing relevance ratings from 140 students; 337 observations were excluded because of missing relevance ratings and/or missing AI-literacy data. Between-student variability in overall relevance ratings was substantial, with a random-intercept variance of *σ*^*2*^ = 2.17, *SD* = 1.47.

Self-reported attitudes towards AI were not significantly associated with overall perceived relevance ratings, *b* = 0.01, *SE* = 0.13, *z* = 0.05, *p* =.096. Attitudes towards AI did not significantly moderate the association between the presented label and perceived relevance, *b* = −0.08, *SE* = 0.05, *z* = −1.50, *p* =.134. Accordingly, there was no evidence that students with more positive attitudes towards AI responded differently to AI-generated versus human-generated labels when evaluating question relevance. The model included 5,463 non-missing relevance ratings from 140 students; 337 observations were excluded because of missing relevance ratings and/or missing attitudes towards AI data. Between-student variability in overall relevance ratings was substantial, with a random-intercept variance of *σ*^*2*^ = 2.20, *SD* = 1.48.

AI literacy was also not significantly associated with perceived quality of MCQ wording ratings, *b* = −0.20, *SE* = 0.14, *z* = −1.49, *p* =.137. AI literacy did not significantly moderate the association between the presented label and perceived quality of wording, *b* = 0.06, *SE* = 0.05, *z* = 1.16, *p* =.247. Accordingly, there was no evidence that students with better AI literacy responded differently to AI-generated versus human-generated labels when evaluating quality of wording. The model included 5,469 non-missing relevance ratings from 140 students; 331 observations were excluded because of missing quality of wording ratings and/or missing AI-literacy data. Between-student variability in overall relevance ratings was substantial, with a random-intercept variance of *σ*^*2*^ = 2.31, *SD* = 1.52.

Attitudes towards AI were also not significantly associated with perceived quality of MCQ wording ratings, *b* = 0.14, *SE* = 0.14, *z* = 1.00, *p* =.316. Attitudes towards AI did not significantly moderate the association between the presented label and perceived quality of wording, *b* = −0.02, *SE* = 0.05, *z* = −0.44, *p* =.660. Accordingly, there was no evidence that students with more positive attitudes towards AI responded differently to AI-generated versus human-generated labels when evaluating quality of wording. The model included 5,469 non-missing relevance ratings from 140 students; 331 observations were excluded because of missing quality of wording ratings and/or missing attitudes towards AI data. Between-student variability in overall relevance ratings was substantial, with a random-intercept variance of *σ*^*2*^ = 2.33, *SD* = 1.53.

## Discussion

### Discussion of the Findings

Our findings suggest that both perceived relevance and perceived quality of wording were significantly influenced by the label. MCQs labelled “ChatGPT-generated” were rated as less relevant to students’ own learning and as less well worded than MCQs labelled “human-authored.” In addition, with regard to perceived relevance, MCQs that had actually been developed by a human medical educator were rated as less relevant than those that had actually been generated by AI. This effect was also reflected in the significant interaction between the label and the actual origin of the items.

Overall, these findings are consistent with both the classical findings of research on algorithm aversion [17, 27] and more recent research on anti-AI bias and AI aversion [18, 28, 29], which generally indicate a devaluation of algorithmic or AI-based recommendations and content.

Although the difference was statistically significant, it should be noted that both AI-generated and human-authored items received very favorable ratings with respect to relevance (mean ratings ranging from 5.64 to 6.00 on the 7-point Likert-scale) and quality of wording (mean ratings ranging from 5.44 to 5.82). Accordingly, the practical significance of these differences should be interpreted as considerably more limited. Given the high ratings, a ceiling effect may even be present, as most students rated the relevance and quality of wording with 6 or 7 points.

One possible explanation for the apparent devaluation of the items that were actually authored by humans is that the ChatGPT-generated items were substantially easier than the human-authored MCQs. Accordingly, students may have perceived the easier items as less threatening to their self-esteem and therefore evaluated them more positively overall, which may in turn have enhanced their ratings of perceived relevance. This explanation is also consistent with the absence of a corresponding devaluation of genuinely human-authored MCQs with regard to quality of wording - possibly because quality of wording is not directly related to students’ own learning progress.

The secondary aim of this study was to explore how students’ AI literacy and attitudes toward AI influence their evaluations of the MCQs. The analysis of the effects of AI literacy and attitudes toward AI was subject to several statistical limitations; therefore, the findings should be interpreted with caution. Nevertheless, we found no evidence that either variable influenced the label effect. The label effect is the primary focus in this context, as it is more directly relevant to assessing anti-AI bias.

This finding is consistent with recent research on anti-AI bias, which has likewise reported inconclusive findings regarding the influence of AI literacy and attitudes toward AI on anti-AI bias effects [21]. One possible explanation for the absence of an effect is that both constructs were assessed using self-report scales. Objective measures of AI literacy might reveal an association between AI literacy and anti-AI bias, as they are less susceptible to social-desirability effects and self-perception biases.

This study builds on the findings of a prior publication by the same research team [7], in which human-generated MCQs had higher discriminatory power than questions made by ChatGPT, although no difference in item difficulty was observed [7]. In the current analysis, ChatGPT-generated items were significantly easier. A likely explanation is that ChatGPT-generated items tended to focus on single topics, while human-generated items incorporated cross-topic references, increasing cognitive load [30]. In addition, compared to the previous study, the list of potential question topics in the present investigation was considerably broader, which likely led the LLM to generate more general questions that lacked depth and, as a result, were not particularly difficult. Ultimately, it is also possible that the LLM prompt used to generate the AI-based MCQs targeted a lower cognitive level than the human-created questions. It is conceivable that the human author of the MCQs placed greater emphasis on application skills rather than on mere recognition. This makes it all the more noteworthy that the discriminatory power of the two question sets was similarly strong.

### Limitations

Several limitations should be noted. This study was conducted with students from a single curriculum at one university, limiting generalizability. Furthermore, the participation was voluntary, potentially introducing self-selection bias. Students with higher academic competence may have been overrepresented. While other studies have shown comparable quality between AI- and human-generated items [11, 31], the ChatGPT prompts used here produced easier questions, which may have been due to the overly broad and abstract list of topics used to construct the prompts.

In addition, the students’ rating of the MCQs showed a ceiling effect. Accordingly, our operationalization may have resulted in not capturing the full range of possible responses across all levels of granularity. Furthermore, item difficulty may have influenced the assessment of MCQs’ relevance. Because the ChatGPT-generated MCQs were easier than the human-authored items, this difference may have produced a halo effect, with students generally viewing the easier MCQs more favorably.

Another potential limitation of this study is that the preparatory examination served as a formative assessment. It is conceivable that medical students might evaluate LLM-generated questions much more critically in the context of high-stakes, summative examinations.

Finally, our study used a within-subjects design, in contrast to studies by other authors on anti-AI bias, which have predominantly employed between-subjects designs [32–35]. The few studies that used within-subjects designs often reported less clear-cut findings and a weaker anti-AI bias or algorithm-aversion effect [21, 36]. Studies using within-subjects designs have generally yielded less pronounced findings regarding anti-AI bias, possibly because the direct comparison between AI-generated and human-authored content makes it apparent that the quality of the supposedly diametrically opposed sources differs little.

### Practical Implications

This study provides evidence of a statistically significant anti-AI bias among students toward AI-generated MCQs. However, the practical significance of this effect is questionable, as both the relevance and quality of wording of the AI-generated questions were rated very favorably, albeit slightly lower than those of the human-authored questions. Accordingly, the statistical evidence of anti-AI bias should not necessarily discourage the use of AI to generate MCQs.

Rather, these findings suggest that, in addition to more important objective measures such as item difficulty and discrimination, softer factors such as anti-AI bias must also be considered when using generative AI in formative education. Medical schools should be aware of the potential relevance of anti-AI bias, but should not overemphasize it, as the real-world effects of this bias are likely to be limited.

## Conclusion

Students rated MCQs labelled as “ChatGPT-generated” as slightly less relevant and less well worded than those labelled as “human-authored,” indicating a statistically significant but limited anti-AI bias. However, ratings for both types of MCQs were highly favorable, suggesting that the practical relevance of this bias may be modest. Although AI-generated items were easier than human-authored items, their discriminatory power was comparable, supporting the cautious use of generative AI for formative assessment while emphasizing the importance of expert review and objective item-quality criteria.

## Supplementary Information

Below is the link to the electronic supplementary material.

Supplementary Material 1 (DOCX 3.10 MB)

## Data Availability

All data were generated in the course of conducting this study. No external data sources were used. The anonymized data set as well as the R-Code can be obtained by the corresponding author upon
