# Supplementary material for: Algorithm Aversion Towards AI-Generated Multiple-Choice Questions
Source: Med Sci Educ. 2026 Aug 28;36(6):2945–53. doi: 10.1007/s40670-026-02866-6 (PMC13759865; doi:10.1007/s40670-026-02866-6)
Supplement: Supplementary file 1 [file 40670_2026_2866_MOESM1_ESM.docx]

## Supplementary Material S1: List of MCQ topics

1. Ligand-gated ion channels
2. Foundations of the resting membrane potential and the action potential
3. Propagation of electrical signals along neuronal membranes
4. Chemical synaptic transmission
5. Electrical synaptic transmission
6. Contraction of striated (skeletal) muscle
7. Structure, function, and contraction of smooth muscle
8. The somatovisceral sensory system
9. Nociception and pain
10. Vestibular sensation and the perception of body position and movement
11. Vision and eye movements
12. Retinal structure, signal acquisition, and signal processing
13. Gustation and olfaction
14. Molecular mechanisms of sensory transduction
15. Sensory thresholds
16. Functional properties of somatosensory neurons
17. Mechanoreception and proprioception
18. Thermoreception
19. Sound conduction to the inner ear and cochlear transduction
20. Signal transformation from sensory cells to the auditory nerve

## Supplementary Material S2: ChatGPT Prompt

### Original German Version:

Erstelle eine Multiple-Choice-Frage mit 5 Antwortmöglichkeiten und nur einer richtigen Antwort. Die Frage richtet sich an fortgeschrittene Medizinstudierende, die eine Abschlussprüfung im Fach Neurophysiologie schreiben. Die Fragenschwierigkeit soll mittel bis hoch sein. Das Thema der Frage ist [spezifisches Thema]. Die Frage soll sich auf [Lernziel] beziehen. Dabei müssen die Begriffe nicht zwingend in jeder Antwortmöglichkeit vorkommen. Achte darauf, dass sich die Antwortmöglichkeiten nicht gegenseitig ausschließen – das heißt, unterschiedliche Antwortoptionen dürfen keine sich direkt widersprechenden Aussagen enthalten. Verzichte in den Antwortoptionen auf absolute Formulierungen wie "immer", "ausschließlich" oder "nie". Gib am Ende die richtige Antwort an.

### English Translation:

Create a multiple-choice question with 5 answer options and only one correct answer. The question is aimed at advanced medical students preparing for a final examination in neurophysiology. The difficulty level of the question should be medium to high. The topic of the question is [specific topic]. The question should relate to [learning objective]. It is not necessary for the key terms to appear in every answer option. Ensure that the answer options do not mutually exclude each other – that is, different answer options must not contain directly contradictory statements. Avoid using absolute terms such as 'always', 'exclusively', or 'never' in the answer options. At the end, indicate the correct answer.

## Supplementary Material S3: Unaltered original exam, validated questionnaires in German and English

### Unaltered original version

*[Note: Introduction to the mock exam, in German]*

**Einführungstext**

Sehr geehrte Teilnehmende,

dieser Fragebogen besteht aus drei Teilen. Im ersten Teil finden Sie 20 Übungsklausurfragen, die von einer medizinischen Lehrperson der Neurophysiologie in Bonn erstellt wurden. Im zweiten Teil finden Sie 20 Fragen, welche von einem KI-basierten Sprachmodell (OpenAI's ChatGPT) generiert wurden. Im letzten Teil stellen wir Ihnen einige Fragen zu Ihrer Kompetenz und Ihren Einstellungen im Bereich künstlicher Intelligenz.

Für die Bearbeitung haben Sie maximal 75 Minuten Zeit.

Die Übungklausur soll einen Beitrag leisten, Sie auf die tatsächliche Neurophysiologie-Klausur vorzubereiten. Gleichzeitig dient die Durchführung der Übungsklausur als Grundlage für eine medizindidaktische Studie, welche vom Institut für Medizindidaktik durchgeführt wird.

Bitte lesen Sie daher die angehangene Probandeninformation sorgfältig. Sollten Sie **NICHT** wünschen, dass Ihre anonymen Daten für die medizindidaktische Studie genutzt werden, können Sie dies im Folgenden angeben. Wenn Sie mit der Datenverarbeitung im Rahmen der Studie einverstanden sind, lassen Sie dieses Feld bitte LEER.

O Ich möchte NICHT, dass meine Daten für die medizindidaktische Studie verwendet werden.

Sie haben die Möglichkeit, ein individuelles Feedback über Ihre Leistung in der Übungsklausur zu erhalten. Damit Ihre Daten anonym bleiben, erhalten Sie zusammen mit dieser Klausur einen individuellen Code.

Bitte übertragen Sie diesen Code in das folgende Feld, wenn Sie ein Feedback zu Ihrer Leistung erhalten möchten.

Bitte bewahren Sie den Code gut auf, da es die einzige Möglichkeit ist, Ihre persönliche Leistung nachvollziehen zu können.

Wie lautet Ihr individueller Code? _ _ _

*[Note: Question block 1, labeled as “Questions developed by a medical teacher, in German]*

**Teil 1: Fragen von Medizindozierenden erstellt**

1.

Welche Aussage trifft am ehesten auf elektrische Synapsen im menschlichen Nervensystem zu?

A. Elektrische Synapsen vermitteln Signale ausschließlich über ligandgesteuerte Ionenkanäle
B. Die Signalübertragung an elektrischen Synapsen erfolgt verzögert, da sie von sekundären Botenstoffen abhängig ist
C. Elektrische Synapsen sind auf die Übertragung hemmender Signale beschränkt
D. Elektrische Synapsen ermöglichen eine bidirektionale Signalübertragung über gap junctions
E. Die Übertragung an elektrischen Synapsen kann durch präsynaptische Rezeptoren gezielt moduliert werden

2.

Welche Aussage zur glatten Muskulatur ist am ehesten zutreffend?

A. Die Kontraktion glatter Muskulatur erfolgt ausschließlich über eine neuronale Innervation durch motorische Endplatten
B. Die Phosphorylierung der leichten Myosinketten durch die Myosin-Leichtketten-Kinase (MLCK) ist entscheidend für die Auslösung der Kontraktion
C. Im Gegensatz zur quergestreiften Muskulatur ist in glatten Muskelzellen kein Aktin und Myosin vorhanden
D. Glatte Muskulatur kontrahiert schneller als Skelettmuskulatur, da Calcium direkt an das Troponin-C bindet
E. Die glatte Muskulatur weist keine regulatorischen Mechanismen zur Beeinflussung der Kontraktionskraft auf

3.

Welche Aussage beschreibt am zutreffendsten den Mechanismus chemischer Synapsen im zentralen Nervensystem?

A. Die Transmitterfreisetzung erfolgt durch Exozytose, sobald sich spannungsabhängige Kaliumkanäle öffnen
B. Chemische Synapsen leiten elektrische Signale bidirektional über gap junctions weiter
C. Der Calciumeinstrom in die präsynaptische Endigung ist entscheidend für die Fusion synaptischer Vesikel mit der Membran
D. Die postsynaptische Antwort hängt ausschließlich von der Anzahl der ausgeschütteten Vesikel ab
E. Neurotransmitter gelangen aktiv durch ATP-abhängige Transporter in den synaptischen Spalt

4.

Durch welche Änderung der zytosolischen Ionen-Konzentration wird die Permeabilität von Gap junctions (auch ohne begleitende Depolarisation) am wahrscheinlichsten herabgesetzt?

A. Zunahme der K⁺-Konzentration
B. Abnahme der Na⁺-Konzentration
C. Zunahme der Cl⁻-Konzentration
D. Zunahme der HCO₃⁻-Konzentration
E. Zunahme der H⁺-Konzentration

5.

Welche Aussage beschreibt am zutreffendsten die Funktion des vestibulären Systems bei der Lage- und Bewegungsempfindung des Menschen?

A. Die Endolymphe der Bogengänge bewegt sich bei Kopfrotation träge und lenkt die Cupula aus, was eine Reizung der Haarzellen verursacht
B. Die Maculaorgane (Utriculus und Sacculus) sind für die Detektion von Rotationsbewegungen um die Körperachsen verantwortlich
C. Die Haarzellen des vestibulären Systems feuern nur dann Aktionspotentiale, wenn sie durch Reizung vollständig depolarisiert werden
D. Die Bogengänge registrieren primär lineare Beschleunigungen des Körpers in vertikaler Richtung
E. Die Informationen aus dem vestibulären Apparat gelangen ausschließlich über den Tractus spinocerebellaris in das Kleinhirn

6.

Bei welcher der Sinnesempfindungen kommt es bei konstanter Reizstärke am wahrscheinlichsten zu einer ausgeprägten peripheren Sensibilisierung?

A. Berührungsempfindung durch Reizung von Meissner-Körperchen auf der Fußsohle
B. Geruchsempfindung durch Schwefelwasserstoff im Bereich des Riechepithels
C. Geschmacksempfindung durch Kochsalzlösung auf der Zunge
D. Schmerzempfindung durch eine Entzündung im Bereich der Fingerbeere
E. Vibrationsempfindung durch Reizung von (Vater-)Pacini-Körperchen auf der Handinnenfläche

7.

Welche Aussage trifft am ehesten auf die neurophysiologischen Grundlagen von Nozizeption und Schmerz zu?

A. Nozizeptoren adaptieren rasch und ermöglichen dadurch eine effektive Unterdrückung chronischer Schmerzreize
B. Substanz P wirkt als Neurotransmitter in der zentralen Schmerzverarbeitung und fördert die Weiterleitung nozizeptiver Reize
C. Die Erregung von Nozizeptoren erfolgt ausschließlich durch mechanische Reize
D. A-δ-Fasern vermitteln primär den langsam einsetzenden, dumpfen Schmerz
E. Die Hemmung nozizeptiver Signale erfolgt ausschließlich auf kortikaler Ebene über inhibitorische Interneurone

8.

Welche Aussage beschreibt am zutreffendsten die Rolle von Propriozeptoren im menschlichen Körper?

A. Propriozeptoren befinden sich ausschließlich in der Haut und messen dort Vibrationen zur Lagewahrnehmung
B. Propriozeptive Informationen werden über schnell leitende, stark myelinisierte Fasern zum ZNS übermittelt
C. Golgi-Sehnenorgane registrieren Änderungen der Muskeldehnung und fördern durch Aktivierung die Muskelkontraktion
D. Muskelspindeln detektieren Veränderungen der Muskelspannung und lösen monosynaptische Reflexe aus
E. Propriozeption ist ein ausschließlich kortikaler Prozess, der unabhängig von spinalen Verschaltungen funktioniert

9.

Welcher der folgenden Vorgänge wird durch eine schnelle passive Dehnung eines Skelettmuskels am wahrscheinlichsten ausgelöst?

A. Abnahme der Aktionspotentialfrequenz in Ia-Fasern desselben Muskels
B. Abnahme der Aktionspotentialfrequenz in Ib-Fasern desselben Muskels
C. Acetylcholin-Freisetzung an motorischen Endplatten desselben Muskels
D. Erschlaffung desselben Muskels
E. Kontraktion des antagonistischen Muskels

10.

Ein Patient erhält an einem Auge Augentropfen, die muscarinische Acetylcholin-Rezeptoren aktivieren.

Welche Folge hat dies am wahrscheinlichsten?

A. fehlende konsensuelle Lichtreaktion des unbehandelten Auges
B. Tonussteigerung der äußeren Augenmuskeln
C. verminderte Fähigkeit zur Nahakkommodation
D. verminderte Tränensekretion
E. verminderte Weitstellung der Pupille im Dunkeln

11.

Ryanodin-Rezeptoren vom Typ I in Skelettmuskelfasern…

A. sind Anionen-Kanäle
B. sind G-Protein-gekoppelte Rezeptoren
C. sind in der Plasmamembran der T-Tubuli lokalisiert
D. sind nicotinerge Acetylcholin-Rezeptoren
E. stehen in engem Kontakt mit spannungsabhängigen Dihydropyridin-Rezeptoren

12.

Um welchen Faktor muss der Schalldruck im Vergleich zum Gesunden mindestens erhöht werden, damit ein Patient mit 60 dB SPL Hörverlust bei 1000 Hz einen Ton wahrnehmen kann?

A. 60
B. 100
C. 1 000
D. 6 000
E. 60 000

13.

An olfaktorische Rezeptoren (ORs) bindende Geruchsstoffe entfalten ihre Wirkung in den Sinneszellen der Regio olfactoria typischerweise über...

A. aktivierte MAP-Kinase
B. cAMP
C. cGMP
D. Inositol-1,4,5-trisphosphat
E. phosphorylierte STAT-Dimere

14.

Welche Aussage beschreibt am zutreffendsten den Begriff der sensorischen Schwelle in der Neurophysiologie?

A. Die sensorische Schwelle ist der Reizbereich, bei dem ein Rezeptor maximal feuert
B. Die absolute Schwelle ist definiert als die minimale Reizintensität, die eine gerade noch wahrnehmbare Empfindung auslöst
C. Sensorische Schwellen sind konstant und werden durch zentrale Mechanismen nicht beeinflusst
D. Eine niedrige sensorische Schwelle bedeutet, dass ein starker Reiz nötig ist, um eine Empfindung auszulösen
E. Unterschiedsschwellen beziehen sich auf die Fähigkeit, verschiedene Sinnesmodalitäten voneinander zu unterscheiden

15.

Welche Aussage beschreibt am zutreffendsten einen Aspekt des somatoviszeralen sensorischen Systems?

A. Schmerzempfindungen aus inneren Organen können durch Konvergenz auf Rückenmarksebenen als somatischer Schmerz fehlgedeutet werden
B. Somatosensorische Informationen aus der Haut erreichen das Rückenmark über motorische Vorderwurzelfasern
C. Viszerale Afferenzen werden ausschließlich über A-β-Fasern mit hoher Leitungsgeschwindigkeit zum ZNS geleitet
D. Das somatoviszerale sensorische System leitet ausschließlich bewusste Empfindungen an den primären somatosensorischen Kortex
E. Propriozeptive Signale aus Muskeln und Gelenken sind funktionell dem viszeralen System zuzuordnen

16.

Welche Aussage beschreibt am zutreffendsten den Ablauf der Signalaufnahme und -verarbeitung in der Retina?

A. Photorezeptoren erzeugen Aktionspotenziale in Abhängigkeit von der Lichtintensität und leiten diese direkt an den Sehnerv weiter
B. Die Depolarisation der Photorezeptoren bei Lichteinfall führt zur vermehrten Freisetzung von Glutamat an Bipolarzellen
C. Ganglienzellen reagieren auf Lichtreize, indem sie ihren Neurotransmitter über elektrische Synapsen an Photorezeptoren zurückkoppeln
D. Horizontalzellen vermitteln laterale Inhibition und tragen zur Kontrastverstärkung im rezeptiven Feld bei
E. Die Umwandlung von Licht in ein elektrisches Signal (Phototransduktion) erfolgt in der inneren Körnerschicht der Retina

17.

Beim intermodalen Intensitätsvergleich...

A. wird die Empfindungsstärke in eine Wahrnehmungsstärke überführt
B. wird die Empfindungsstärke in eine Nominalskala überführt
C. wird die Unterschiedsschwelle in Abhängigkeit vom Ausgangsreiz gemessen
D. wird die Empfindungsstärke in eine Rationalskala überführt
E. wird die Absolutschwelle ermittelt

18.

Welche Aussage beschreibt am zutreffendsten die Funktion des thalamokortikalen nozizeptiven Systems und endogener Schmerzkontrollmechanismen?

A. Nozizeptive Signale erreichen über spezifische thalamische Kerne kortikale Areale wie den primären somatosensorischen Kortex
B. Der Thalamus dient ausschließlich der motorischen Integration und ist nicht an der Verarbeitung nozizeptiver Signale beteiligt
C. Endogene Schmerzkontrolle erfolgt ausschließlich durch hemmende Interneurone im Hinterhorn ohne Beteiligung supraspinaler Strukturen
D. Das periaquäduktale Grau hemmt die Schmerzleitung durch direkte Inhibition von C-Fasern im peripheren Nervensystem
E. Die kortikale Schmerzwahrnehmung ist unabhängig von der Projektion thalamischer Neurone in den Gyrus cinguli

19.

Für die Entstehung welcher der Schmerzkomponenten sind intakte Ventrobasalkerne des Thalamus notwendig?

A. affektive Komponente
B. psychomotorische Komponente
C. sensorisch-diskriminative Komponente
D. spinalmotorische Komponente
E. vegetative Komponente

20.

Welche Aussage zur Akkommodationsbreite eines Patienten mit Nahpunkt bei 10 cm und Fernpunkt bei 50 cm trifft am ehesten zu?

A. Aus den Angaben kann seine Akkommodationsbreite nicht bestimmt werden
B. Seine Akkommodationsbreite beträgt 2 dpt
C. Seine Akkommodationsbreite beträgt 2,5 dpt
D. Seine Akkommodationsbreite beträgt 8 dpt
E. Seine Akkommodationsbreite beträgt 10 dpt

*[Note: Question block 2, labeled as “Questions developed by ChatGPT, in German]*

**Teil 2: Von ChatGPT generiert**

1.

Geben Sie Cl⁻, HCO₃⁻ und K⁺ bezüglich ihrer Stoffmengenkonzentrationen im Extrazellularraum in absteigender Reihenfolge an:

A. Cl⁻ > HCO₃⁻ > K⁺
B. Cl⁻ > K⁺ > HCO₃⁻
C. HCO₃⁻ > Cl⁻ > K⁺
D. K⁺ > Cl⁻ > HCO₃⁻
E. K⁺ > HCO₃⁻ > Cl⁻

2.

Welche Aussage beschreibt am zutreffendsten die Schallleitung zum Innenohr?

A. Die Schwingungen des Steigbügels werden direkt über die Reissner-Membran an die Haarzellen der Cochlea weitergeleitet
B. Das Trommelfell verstärkt die Schallenergie durch Resonanz mit der Perilymphe der Scala tympani
C. Die Hebelwirkung der Gehörknöchelchen transformiert Luftschall in eine Druckwelle im Innenohr, was zur Auslenkung der Basilarmembran führt
D. Die Gehörknöchelchenkette überträgt mechanische Schwingungen vom ovalen Fenster zum runden Fenster
E. Die Schallleitung zum Innenohr ist ausschließlich bei hohen Frequenzen aktiv; tiefe Töne werden durch Luftleitung im Mittelohr überbrückt

3.

In welcher der folgenden Antwortmöglichkeiten ist der Neurotransmitter zwischen präsynaptischem Neuron und Zielzelle typischerweise richtig zugeordnet?

A. Ia-Muskelspindelafferenz → GABA → α-Motoneuron
B. II-Muskelspindelafferenz → Glycin → inhibitorisches Ia-Interneuron
C. inhibitorisches Ia-Interneuron → Glutamat → α-Motoneuron
D. Renshaw-Zelle → Glutamat → α-Motoneuron
E. α-Motoneuron → Acetylcholin → Renshaw-Zelle

4.

Ein älterer emmetroper Patient hat noch eine Akkommodationsbreite von 2 dpt und trägt eine Lesebrille von +1 dpt. (Der Abstand der Brille vom Auge ist vernachlässigbar klein.)

Etwa in welchem Abstand vom Auge befindet sich dann sein Nahpunkt mit Brille?

A. 10 cm
B. 20 cm
C. 33 cm
D. 50 cm
E. 100 cm

5.

Bei einem drei Wochen alten Säugling soll überprüft werden, ob das Hörvermögen intakt oder durch eine Störung der Schallleitung bzw. des Innenohrs vermindert ist.

Welches der folgenden Verfahren ist hierfür am ehesten geeignet?

A. Messung (evozierter) otoakustischer Emissionen
B. Rinne-Versuch
C. tonaudiometrische Bestimmung der Hörschwellen über einen auf das Mastoid aufgesetzten Tongeber (Knochenleitungshörer)
D. tonaudiometrische Bestimmung der Hörschwellen über Kopfhörer
E. Weber-Versuch

6.

Welche Aussage beschreibt am zutreffendsten die Signaltransformation von der Sinneszelle zum Hörnerv im auditorischen System?

A. Die mechanische Auslenkung der Haarzellen führt zur Öffnung spannungsabhängiger Calciumkanäle, was die Transmitterausschüttung an afferente Nervenfasern ermöglicht
B. Die inneren Haarzellen generieren Aktionspotenziale, die über chemische Synapsen direkt in den Nervus vestibularis überführt werden
C. Die äußeren Haarzellen übermitteln die eigentliche Hörinformation an den Hörnerv, während die inneren Haarzellen hauptsächlich der Verstärkung dienen
D. Die Signalübertragung von den Haarzellen auf die afferenten Fasern erfolgt ausschließlich durch elektrische Synapsen über Gap Junctions
E. Der Hörnerv ist direkt mit der Tektorialmembran verbunden, wodurch mechanische Reize ohne synaptische Umwandlung weitergeleitet werden

7.

Welche Aussage beschreibt am zutreffendsten die neurophysiologischen Grundlagen der Riechnerven (Nervus olfactorius)?

A. Der Nervus olfactorius besteht aus Axonen bipolarer primärer Sinneszellen, die durch die Lamina cribrosa ziehen
B. Die Riechzellen sind sekundäre Sinneszellen mit chemisch aktivierten Mechanorezeptoren
C. Die Axone der Riechzellen ziehen direkt zum Thalamus, bevor sie den primären olfaktorischen Kortex erreichen
D. Der olfaktorische Signalweg zeigt im Gegensatz zu anderen Sinnessystemen eine vollständige Kreuzung in der Medulla oblongata
E. Die Transduktion des Geruchsreizes basiert auf einer direkten Depolarisation durch Na⁺-Kanäle ohne G-Protein-vermittelte Prozesse

8.

Welche Aussage zu Blicksakkaden trifft typischerweise zu?

A. Die Bewegungsamplitude einer Sakkade beträgt maximal 10 Grad
B. Sakkaden erfolgen immer reflektorisch
C. Sakkaden werden während der Ausführung durch visuelle Rückkopplung präzise korrigiert
D. Typisch für eine Sakkade sind zwei in entgegengesetzter Richtung laufende Bulbusbewegungen mit unterschiedlicher Geschwindigkeit
E. Während der Sakkade wird der Seheindruck kurz eingeschränkt bzw. unterbrochen

9.

Welche Aussage beschreibt am zutreffendsten die physiologischen Grundlagen der Thermorezeption?

A. Die Aktivität thermosensitiver Ionenkanäle wie TRPV1 oder TRPM8 bestimmt das Antwortverhalten der Thermorezeptoren
B. Kaltrezeptoren sind primär an Aβ-Fasern gekoppelt und feuern nur bei Temperaturen unter 0 °C
C. Wärme- und Kaltrezeptoren adaptieren vollständig, sodass anhaltende Temperaturreize nicht mehr wahrgenommen werden
D. Thermorezeptoren reagieren ausschließlich auf Gewebeschäden und vermitteln daher nur nozizeptive Signale
E. Thermorezeptive Informationen werden direkt im primär motorischen Cortex verarbeitet, um rasche Reaktionen zu ermöglichen

10.

Welche Aussage zur elektromyographischen Registrierung trifft bei schrittweiser Steigerung der Reizstärke (35 V bis 95 V) am wahrscheinlichsten zu?

A. Bei hohen Reizstärken wird die Amplitude der H-Welle größer als die Amplitude der M-Welle
B. Die Amplitude der H-Welle bleibt mit steigender Reizstärke gleich groß
C. Die Amplitude der M-Welle nimmt mit steigender Reizstärke ab
D. Die maximale Amplitude der H-Welle ist kleiner als die maximale Amplitude der M-Welle
E. Die M-Welle erscheint bereits bei geringeren Reizstärken als die H-Welle

11.

Welche Aussage beschreibt am zutreffendsten die neuronalen Grundlagen des Sehens und der Augenbewegungen?

A. Photorezeptoren feuern Aktionspotentiale, sobald sie durch Licht hyperpolarisiert werden
B. Der Tractus opticus enthält Nervenfasern, die aus beiden Augen stammen und zum Corpus geniculatum laterale projizieren
C. Die Steuerung sakkadischer Augenbewegungen erfolgt ausschließlich durch den Nucleus pretectalis
D. Der nervus opticus leitet visuelle Informationen beider Hemifelder zum ipsilateralen Thalamus
E. Die laterale Hemmung im Bereich der Retina erfolgt über amakrine Zellen zur Kontrastverstärkung

12.

Welche Aussage beschreibt am zutreffendsten einen zentralen Aspekt der Kontraktion quergestreifter Muskeln?

A. Die Freisetzung von Calciumionen aus dem Sarkolemm initiiert die Kontraktion durch Bindung an Troponin C
B. Die Gleitfilamenttheorie besagt, dass sich während der Kontraktion die Länge der Aktin- und Myosinfilamente verringert
C. ATP wird ausschließlich zur Rückführung von Calcium in das Sarkolemm benötigt, nicht jedoch für den Querbrückenzyklus
D. Die Myosinköpfchen lösen sich vom Aktinfilament, sobald sie ADP und anorganisches Phosphat binden
E. Die Depolarisation des T-Tubulus-Systems führt über spannungsabhängige Dihydropyridinrezeptoren zur Aktivierung der Ryanodinrezeptoren

13.

Welche Aussage beschreibt am zutreffendsten einen molekularen Mechanismus der Reiztransduktion in Sinneszellen?

A. Bei der auditorischen Transduktion öffnen sich spannungsabhängige Kaliumkanäle durch Depolarisation der Haarzellen
B. In Riechzellen führt die Aktivierung eines G-Protein-gekoppelten Rezeptors zur Öffnung von Ca²⁺-Kanälen über cAMP-vermittelte Signalwege
C. Die Transduktion in Photorezeptoren basiert auf einer lichtinduzierten Depolarisation durch Natriumeinstrom
D. Mechanosensitive Ionenkanäle in Haarzellen werden durch ATP aktiviert, das aus benachbarten Zellen freigesetzt wird
E. Die molekulare Transduktion in Geschmackszellen erfolgt ausschließlich durch direkte Ionenkanäle ohne Second Messenger

14.

Welche Aussage zu β₂-Adrenorezeptoren auf glatten Muskelzellen trifft am ehesten zu?

A. erhöht die Aktivität der Myosin-leichte-Ketten-Kinase (MLCK)
B. erhöht die Kontraktionskraft
C. führt zur vermehrten Öffnung von Ca²⁺-Einstrom aus dem endoplasmatischen Retikulum über IP₃-gesteuerte Ca²⁺-Kanäle
D. vermittelt direkt eine Depolarisation durch Einstrom von Ca²⁺
E. verstärkt die Aktivität der sarkoplasmatischen Na⁺/Ca²⁺-Pumpe

15.

Welche der folgenden Aussagen beschreibt am zutreffendsten die elektrophysiologischen Grundlagen des Ruhemembran- und Aktionspotentials in Nervenzellen?

A. Während des Ruhezustands ist die Permeabilität der Zellmembran für Kaliumionen höher als für Natriumionen
B. Das Aktionspotential wird durch die gleichzeitige Öffnung von spannungsabhängigen Natrium- und Kaliumkanälen ausgelöst
C. Das Ruhemembranpotential entsteht primär durch den Einstrom von Natriumionen durch spannungsabhängige Natriumkanäle
D. Die Repolarisation des Aktionspotentials erfolgt hauptsächlich durch einen Natriumausstrom über die Natrium-Kalium-Pumpe
E. Das Erreichen des Schwellenpotentials hängt ausschließlich von der Aktivität ligandgesteuerter Ionenkanäle ab

16.

Welche Aussage zur Fortleitung elektrischer Signale entlang der neuronalen Membran ist am ehesten zutreffend?

A. Die kontinuierliche Fortleitung elektrischer Signale ist ausschließlich auf myelinisierte Axone beschränkt
B. In nicht-myelinierten Axonen erfolgt die Fortleitung elektrischer Signale durch Depolarisation benachbarter internodaler Membranbereiche
C. Die Geschwindigkeit der Erregungsleitung ist unabhängig vom Axondurchmesser, aber abhängig von der Dichte spannungsabhängiger Kaliumkanäle
D. Myelinisierung reduziert den Membranwiderstand und fördert dadurch die schnellere Fortleitung elektrischer Signale
E. Bei der saltatorischen Erregungsleitung werden Aktionspotentiale nur an Ranvier-Schnürringen generiert

17.

Welche Aussage zur Cochlea trifft am besten zu?

A. Der Absolutbetrag der Potentialdifferenz zwischen dem Endo- und dem Perilymphraum liegt bei etwa 30 mV
B. Der Absolutbetrag der Potentialdifferenz zwischen Intrazellulärraum einer äußeren Haarzelle und dem Endolymphraum liegt bei etwa 150 mV
C. Die Flüssigkeit in der Scala media ähnelt in ihrer Ionen-Konzentration mehr der interstitiellen als der zytosolischen Flüssigkeit
D. In der Scala media befindet sich Perilymphe
E. In der Scala vestibuli befindet sich Endolymphe

18.

Dicke Axone zeigen im Vergleich zu dünneren Axonen...

A. ein größeres Aktionspotential bei extrazellulärer elektrischer Reizung erst bei größeren Reizen
B. Aktionspotentiale
C. einen größeren Längswiderstand
D. einen kleineren Membranwiderstand am Ruhemembranpotential
E. eine kleinere Membranleitfähigkeit am Ruhemembranpotential

19.

Welche der folgenden Zellen phagozytieren die Endabschnitte der Außenglieder der Photorezeptoren?

A. amakrine Zellen
B. Bipolarzellen
C. Ganglienzellen
D. Horizontalzellen
E. Pigmentepithelzellen

20.

Welche Aussage beschreibt am zutreffendsten das Zusammenspiel zwischen dem nozizeptiven System und der subjektiven Schmerzempfindung?

A. Die Projektion nozizeptiver Signale ins limbische System erklärt die emotionale Komponente der Schmerzwahrnehmung
B. Das nozizeptive System endet im Rückenmark, da die bewusste Wahrnehmung von Schmerz dort stattfindet
C. Die subjektive Schmerzempfindung wird ausschließlich durch die Intensität des peripheren Reizes bestimmt
D. Die Verarbeitung nozizeptiver Signale ist unabhängig von kortikalen Strukturen wie dem Gyrus cinguli oder dem Inselkortex
E. Das nozizeptive System vermittelt nur akute, aber keine chronischen Schmerzen, da diese rein psychogener Natur sind

*[Note: Final part of the questionnaire, consisting of SNAIL short version and ATTARI-12, both in German]*

**Teil 3: KI-Kompetenzen und Einstellungen gegenüber KI**

**SNAIL (German short version)**

Ich kann…

1.

beschreiben, wie Machine Learning Modelle trainiert, validiert und getestet werden.

2.

erklären, in welchem Verhältnis Deep Learning zu Machine Learning steht.

3.

erklären, wie sich regelbasierte Systeme von Machine Learning Systemen unterscheiden.

4.

erklären, wie KI Anwendungen Entscheidungen treffen

5.

erklären, warum Datenschutz beachtet werden muss, wenn Anwendungen künstlicher Intelligenz entwickelt und verwendet werden.

6.

erklären, warum Datensicherheit beachtet werden muss, wenn Anwendungen künstlicher Intelligenz entwickelt und verwendet werden.

7.

ethische Bedenken rund um künstliche Intelligenz identifizieren.

8.

Risiken beschreiben, welche auftreten können, wenn Systeme künstlicher Intelligenz verwendet werden.

9.

Beispiele aus meinem täglichen Leben (privat oder beruflich) geben, bei denen ich in Kontakt mit künstlicher Intelligenz sein könnte.

10.

Beispiele technischer Anwendungen nennen, die von künstlicher Intelligenz unterstützt werden.

11.

erkennen, ob die Technologien, die ich nutze, von künstlicher Intelligenz unterstützt werden.

12.

beurteilen, ob ein Problem in meinem Fachbereich mit Methoden der künstlichen Intelligenz gelöst werden kann und sollte.

**ATTARI-12 (German version)**

1.

Künstliche Intelligenz wird die Welt verbessern.

2.

Ich habe starke negative Emotionen gegenüber künstlicher Intelligenz.

3.

Ich möchte Technologien nutzen, die auf künstlicher Intelligenz basieren.

4.

Künstliche Intelligenz hat mehr Nachteile als Vorteile.

5.

Ich freue mich auf zukünftige Entwicklungen im Bereich künstliche Intelligenz.

6.

Künstliche Intelligenz bietet Lösungen für viele globale Probleme.

7.

Ich bevorzuge Technologien, die keine künstliche Intelligenz beinhalten.

8.

Ich fürchte mich vor künstlicher Intelligenz.

9.

Ich würde mich eher für eine Technologie mit künstlicher Intelligenz entscheiden als für eine ohne.

10.

Künstliche Intelligenz verursacht eher Probleme, anstatt sie zu lösen.

11.

Wenn ich an künstliche Intelligenz denke, habe ich hauptsächlich positive Gefühle.

12.

Ich möchte mit Technologien, die auf künstlicher Intelligenz beruhen, lieber nichts zu tun haben.

### Original validated SNAIL and ATTARI-12 questionnaires in English

**SNAIL (Short version)**

From: Laupichler, M. C., Aster, A., Haverkamp, N., & Raupach, T. (2023). Development of the “Scale for the assessment of non-experts’ AI literacy”–An exploratory factor analysis. *Computers in Human Behavior Reports, 12*, 100338.

I can…

1. describe how machine learning models are trained, validated, and tested.
2. explain how deep learning relates to machine learning
3. explain how rule-based systems differ from machine learning systems.
4. explain how AI applications make decisions.
5. explain why data privacy must be considered when developing and using artificial intelligence applications.
6. explain why data security must be considered when developing and using artificial intelligence applications.
7. identify ethical issues surrounding artificial intelligence.
8. describe risks that may arise when using artificial intelligence systems.
9. give examples from my daily life (personal or professional) where I might be in contact with artificial intelligence.
10. name examples of technical applications that are supported by artificial intelligence.
11. tell if the technologies I use are supported by artificial intelligence.
12. assess if a problem in my field can and should be solved with artificial intelligence methods.

**ATTARI-12**

From: Stein, J. P., Messingschlager, T., Gnambs, T., Hutmacher, F., & Appel, M. (2024). Attitudes towards AI: measurement and associations with personality. *Scientific Reports, 14*(1), 2909.

1. AI will make this world a better place.
2. I have strong negative emotions about AI. (negative - reverse coded)
3. I want to use technologies that rely on AI.
4. AI has more disadvantages than advantages. (negative - reverse coded)
5. I look forward to future AI developments.
6. AI offers solutions to many world problems.
7. I prefer technologies that do not feature AI. (negative - reverse coded)
8. I am afraid of AI. (negative - reverse coded)
9. I would rather choose a technology with AI than one without it.
10. AI creates problems rather than solving them. (negative - reverse coded)
11. When I think about AI, I have mostly positive feelings.
12. I would rather avoid technologies that are based on AI. (negative - reverse coded)

## Supplementary Material S4: Translation of a Selection of Sample MCQs from the Unaltered German Version into English.

## **ChatGPT-generated MCQs:**

Example MCQ 1:

Which of the following statements most accurately describes the electrophysiological foundations of the resting membrane potential and action potential in neurons?

A) At rest, the cell membrane is more permeable to potassium ions than to sodium ions.

B) The action potential is triggered by the simultaneous opening of voltage-gated sodium and potassium channels.

C) The resting membrane potential is primarily generated by the influx of sodium ions through voltage-gated sodium channels.

D) Repolarization of the action potential occurs mainly through the outward flow of sodium ions via the sodium–potassium pump.

E) Reaching the threshold potential depends exclusively on the activity of ligand-gated ion channels.

Correct answer: A)

Example MCQ 2:

Which of the following statements most accurately describes a central aspect of striated muscle contraction?

A) The release of calcium ions from the sarcolemma initiates contraction by binding to troponin C.

B) The sliding-filament theory states that the length of the actin and myosin filaments decreases during contraction.

C) ATP is required exclusively for the reuptake of calcium into the sarcolemma and not for the cross-bridge cycle.

D) Myosin heads detach from the actin filament once they bind ADP and inorganic phosphate.

E) Depolarization of the T-tubule system activates the ryanodine receptors via voltage-gated dihydropyridine receptors.

Correct answer: E)

**MCQ developed by human medical educator:**

Example MCQ 3:

Which of the following statements regarding β₂-adrenergic receptors on smooth muscle cells is most accurate?

Activation of β₂-adrenergic receptors on the cell membrane of smooth muscle cells…

A) increases the activity of myosin light-chain kinase (MLCK).

B) increases contractile force.

C) promotes the opening of IP₃-gated Ca²⁺ channels, thereby increasing Ca²⁺ influx from the endoplasmic reticulum.

D) directly induces depolarization through Ca²⁺ influx.

E) enhances the activity of the sarcoplasmic Na⁺/Ca²⁺ pump.

Correct answer: C)

Example MCQ 4:

A patient receives eye drops in one eye that activate muscarinic acetylcholine receptors. Which of the following effects is most likely?

A) Absence of the consensual light reflex in the untreated eye.

B) Increased tone of the extraocular muscles.

C) Reduced ability to accommodate for near vision.

D) Reduced tear secretion.

E) Reduced pupillary dilation in darkness.

Correct answer: E)
